# Progression through return-to-sport and return-to-academics guidelines for concussion management and recovery in collegiate student athletes: findings from the Ivy League–Big Ten Epidemiology of Concussion Study

**DOI:** 10.1136/bjsports-2021-104451

**Published:** 2022-04-20

**Authors:** Douglas J Wiebe, Abigail C Bretzin, Bernadette A D'Alonzo, and the Ivy League–Big Ten Epidemiology of Concussion Study Investigators, Russell Fiore

**Affiliations:** 1 Biostatistics, Epidemiology and Informatics, University of Pennsylvania, Philadelphia, Pennsylvania, USA; 2 Penn Injury Science Center, University of Pennsylvania, Philadelphia, PA, USA

**Keywords:** Sports, Sporting injuries, Cohort Studies

## Abstract

**Objective:**

To examine the progression of collegiate student athletes through five stages of a return-to-activity protocol following sport-related concussion (SRC).

**Methods:**

In a multisite prospective cohort study, we identified the frequency of initial 24–48 hours physical and cognitive rest, and the sequence of (1) symptom resolution and return to (2) exertion activity, (3) limited sport, (4) full sport and (5) full academics. In resulting profiles we estimated the likelihood of return to full sport ≤14 days or prolonged >28 days and tested for variability based on timing of the stages.

**Results:**

Among 1715 athletes with SRC (31.6% females), 67.9% had 24–48 hours initial physical and cognitive rest. The median was 6 days to return to full academics, 8 days to symptom resolution and 9 days to exertion. Three profiles emerged; all had the same sport-specific return progression, but varied in the relative timing of full academics. In unadjusted analyses, full academics as the first stage corresponded to the longest time to return to full sport, and initiating exertion the same day as symptom resolution resulted in the shortest time. In adjusted regression analyses, athletes initiating full academics while still symptomatic were 21.5% less likely (95% CI −27.4% to −15.5%) to return to full sport ≤14 days and, analogously, 19.1% more likely (95% CI 13.4% to 24.7%) to have prolonged return >28 days. While additionally controlling for initial rest, sex, symptom count and concussion history, the likelihood of prolonged return >28 days was 37.0% (95% CI 25.2% to 48.8%) in athletes initiating exertion considerably before symptoms resolved (ie, 7+ days), but only 3.6% (95% CI −1.4% to 8.6%) in athletes initiating exertion shortly before achieving symptom resolution (ie, 3–4 days).

**Conclusion:**

We found evidence that sequential progressions were consistent with current recommendations including brief initial rest, and the initiation and relative timing of each stage impacted the final return-to-sport outcome.

## Introduction

Athletic trainers are typically the first providers to identify and evaluate injured athletes with sport-related concussion (SRC) and are integral to return-to-sport (RTS) decision-making and management.^1^ A recent survey of athletic trainers in the USA^2^ found the 2014 National Athletic Trainers’ Association (NATA) position statement^1^ to be the RTS guideline followed most often (61%), followed by the 2017 Consensus in Sport Group (CISG) consensus statement (37%)^3^ and the 2013 CISG consensus statement (16%).^4^ This variability in practice could be expected due to the multilevel governing bodies (eg, institution, athletic conference, state) with evolving policies that athletic trainers and their interdisciplinary concussion management team must adhere to.

Both the CISG and the NATA recommend a graduated progression for RTS after SRC. The 2017 CISG consensus statement recommends a brief period of rest during the acute phase (24–48 hours) followed by a six-stage RTS strategy.^3^ This brief initial rest period, and stage 1 as symptom-limited activity are important updates that distinguish the 2017 CISG consensus statement^3^ from the 2013 CISG consensus statement^4^ and from the 2014 NATA position statement, which recommends athletes be asymptomatic before starting an RTS progression.^1^ Stage 1 entails symptom-limited activity, encouraging athletes to gradually become more active in cognitive and physical activities that do not worsen their symptoms, followed by stages 2–6 that entail light aerobic exercise, sport-specific exercise, non-contact training drills, full-contact practice and return to sport, respectively. In addition, the 2017 CISG consensus statement integrates a graduated return-to-school strategy into stage 1 of the RTS progression, with emphasis placed on children and adolescents, suggesting they should not return to sport until they have successfully returned to school, and that early introduction of symptom-limited physical activity is appropriate. However, there is limited guidance relevant to academic return in collegiate student athletes.

This gap could be informed by examining student athletes’ sequential progression to athletic and academic activity following SRC. The resources in the collegiate setting enable frequent symptom monitoring and active guidance by concussion management teams. We studied a large sample of collegiate student athletes with SRC, profiled the sequence of return stages and tested the influence of initial rest, and the timing of academic and athletic activities, as they relate to time to symptom resolution and full sport.

## Methods

### Study setting

The Ivy League–Big Ten Epidemiology of Concussion Study is a surveillance system and prospective cohort study with the goal to better understand SRC and produce evidence to benefit the health and well-being of student athletes. Participating sites include the 8 Ivy League and 12 of 14 universities in the Big Ten.

### Participant identification

An informed consent procedure is used to recruit student athletes who sustain an SRC or non-sport-related concussion as defined by the most recent CISG consensus statement at the time the SRC occurred.^3 4^ Athletic trainers and research coordinators abstract demographic and clinical information including concussion history from the medical record to document a new concussion and administer outstanding questions to the athlete directly. The present analysis includes data from 2013 to 2020 and is limited to athletes in contact/collision sports,^5^ given that return to full-contact practice is a stage of the RTS progression in the 2017 CISG consensus statement.^3^ The data include symptom burden, measured as whether at any time post-injury the athlete reported experiencing each of the 22 symptoms assessed in the Sport Concussion Assessment Tool (SCAT),^6^ a standardised instrument endorsed by the CISG.^3^

### Measures of initial rest, return-to-academics and return-to-sport

Athletic trainers, paired with members of the multidisciplinary concussion management team, re-initiate and progress student athletes through academic and athletic activities following concussion. This study does not directly evaluate details of the RTS protocols and the extent to which practices vary across study sites; however, each site must comply with the NCAA concussion management checklist.^7^ Instead, each site records whether the athlete had an initial period of 24–48 hours physical rest and cognitive rest immediately post-injury.

Our data collection form asks ‘Did the student progress through the return to play protocol as expected?’ with a binary (yes/no) response to indicate if any stage of the RTS was repeated or incomplete. Also, dates are recorded when each athlete (1) self-reported being asymptomatic (ie, returned to baseline symptoms), and returned to (2) exertion activity, (3) limited sport and (4) full sport, and (5) full academics. Each of these milestones is relevant to one of the stages in the RTS and return-to-academics strategies in the 2017 CISG consensus statement and the 2014 NATA position statement. For example, our ‘exertion activity’ milestones corresponds to either ‘light aerobic exercise’ (stage 2) or ‘sport-specific exercise’ (stage 3) in the 2017 CISG consensus statement; ‘limited sport’ corresponds to either ‘sport-specific exercise’ (stage 3) or ‘non-contact training drills’ (stage 4). We refer to these as return-to-activity stages.

### Profiles describing sequence of return

We determined the sequence in which each athlete reached the five return-to-activity stages. We then classified each athlete according to the ‘profile’ describing their return.

### Outcome identification

The primary outcome was returning to full sport ≤14 days post-injury (ie, yes/no), with prolonged delay to full sport >28 days as a secondary outcome. The timing of these outcomes is based on finding that 50% of student athletes in the Ivy League and Big Ten return to full sport in 14 days, yet one-third require longer than 28 days, making them clinically relevant.^8^ Further, these outcomes are easily translated into weekly increments, to aid in interpretation.

### Statistical analysis

#### Descriptive statistics

We used descriptive statistics to determine the percent of student athletes who had initial 24–48 hours of physical and cognitive rest, and to characterise its relation to symptom burden and to time to symptom resolution. We also calculated descriptive statistics to report the frequency of each return-to-activity profile. We used χ^2^ and Kruskal-Wallis tests to examine whether symptom burden, concussion history or having 24–48 hours initial physical and cognitive rest varied in athletes who followed different profiles. Given the study period spanned years when the CISG consensus statement was updated, we tested whether an initial period of rest, and whether having exertion activity before symptom resolution, became more common after publication of the 2017 statement. Also, we tested whether return-to-activity profiles differed by risk factors for prolonged recovery (ie, sex, concussion history, symptom count) and between the two athletic conferences.

Next, we used Kaplan-Meier curves with log-rank tests to determine whether the time to return to full sport differed by return-to-activity profile as an unadjusted, descriptive analysis. Then, as a way to understand the timing of symptom resolution relative to the timing of the other stages, we used scatterplots to array the data at the athlete level and used descriptive statistics to determine the percent of athletes who reached each stage on the same day, or before or after the day they reached symptom resolution. We then used unadjusted Kaplan-Meier curves to determine how the timing of symptom resolution relative to return to full academics and relative to return to exertion activities related to days to return to full play post-injury.

#### Multivariable logistic regression identifying risk factors for return to full play

Finally, we used multivariable logistic regression to test whether the likelihood of returning to full play ≤14 days varied depending on having initial 24–48 hours rest, first, based on the timing of exertion relative to symptom resolution, and then, on the timing of return to full academics relative to symptom resolution. We modelled initial 24–48 hours rest as a dichotomous variable. Based on the pattern revealed in the scatterplots described above, we modelled the timing of exertion as a categorical variable that classified each athlete according to the number of days between when they started exertion and when they experienced symptom resolution. We binned athletes as starting exertion 1–2, 3–4, 5–6 or 7+ days before symptom resolution or 1–2, 3–4, 5–6 or 7+ days after symptom resolution, which put us in a position to detect threshold effects associated with ‘early or late’ exertion (relative to symptom resolution) and also gave a large sample within each bin to achieve precise effect estimates. We used the same approach to create a categorical variable that classified the timing of return to full academics relative to the timing of symptom resolution. After initial analyses on this categorical variable identified a threshold effect, we reclassified the variable to be dichotomous. Doing so provided a more parsimonious model, and in particular let us treat both return to full academics and initial 24–48 hours rest not only as potential confounders also but as effect modifiers as we examined how the relative timing of return to exertion and symptom resolution related to achieving return to full play ≤14 days. The adjusted logistic regression also included sex, symptom burden, previous concussions, and presence and type of other injury as covariates.

We report the results of the adjusted logistic regression as the predicted likelihood (ie, risk=100×probability) of returning to full sport ≤14 days in the student athlete sample overall, and absolute risk differences (ARD) indicating the estimated difference in the likelihood of returning to full sport ≤14 days in sample subgroups. Model fit was assessed using conventional diagnostics.^9 10^

Most (93.0%) cases had complete data, 6.9% had missing data on time to exertion and 12%–16% had missing data on other variables. We used multiple imputation to avoid bias and imprecision that could result from using listwise deletion or a complete case analysis.^11^ This entailed creating 20 datasets where missing values were imputed,^12^ analysing the datasets simultaneously, and pooling the results while adjusting SEs accordingly. Two-sided p values <0.05% and 95% CIs excluding the null value of 1 for likelihood estimates and excluding the null value of 0 for ARDs were considered statistically significant. Analyses were performed using Stata/MP V.16.1 (College Station, Texas, USA). We used the same approach to model prolonged delay to full sport >28 days as the secondary outcome.

### Sensitivity analyses

Adding covariates for athlete age, years competing in their sport, academic year or time since last concussion in the adjusted logistic regression models did not improve model fit. Return to full sport ≤21 days post-injury was examined in a sensitivity analysis to assess whether the findings were sensitive to the time period chosen as the outcome. We repeated the regressions using casewise deletion to gauge imprecision and the magnitude and direction of bias overcome using multiple imputation.

## Results

### Initial 24–48 hours rest

Table 1 reports that 1715 student athletes sustained an SRC. Over two-thirds (67.9%) had initial 24–48 hours of both physical and cognitive rest post-injury, 23.8% had 24–48 hours of only physical rest and 2.9% had only cognitive rest. Athletes with 24–48 hours initial physical and cognitive rest endorsed more symptoms (median=10, IQR=7–15) than did other athletes (median=7, IQR=5–11, p<0.001) and had longer times to symptom resolution (median=10 days, IQR=5–22 vs 4, IQR=2–10, p<0.001). Having 24–48 hours physical and cognitive rest increased from 66.2% to 71.1% (p<0.05) after the 2017 CISG consensus statement.

**Table 1:** Characteristics of 1715 collegiate student athletes with sport-related concussion

Characteristic		N	%	Median	IQR
**Demographics**					
Sex					
Female		542	31.6		
Male		1173	68.4		
Age, years				20	19–21
Academic year					
Freshman		436	25.5		
Sophomore		527	30.9		
Junior		402	23.6		
Senior		321	18.8		
Fifth year		21	1.2		
Years competing in sport				11	8–14
**Sport**					
Basketball	Women	71	4.1		
	Men	57	3.3		
Field hockey	Women	59	3.4		
Football	Men	579	33.8		
Ice hockey	Women	79	4.6		
	Men	121	7.1		
Lacrosse	Women	48	2.8		
	Men	79	4.6		
Rugby	Women	135	7.9		
	Men	43	2.5		
Soccer	Women	115	6.7		
	Men	91	5.3		
Sprint football	Men	52	3		
Water polo	Women	35	2		
	Men	33	1.9		
Wrestling	Men	118	6.9		
**Activity during injury**					
Competition		921	54		
Practice		704	41.2		
Scrimmage		65	3.8		
Skills instruction/strength and conditioning		17	1		
**Concussion history**					
Previous concussions, n					
0		838	48.9		
1		464	27.1		
2		230	13.4		
3		115	6.7		
4 or more		68	3.9		
**24–48 hours initial rest immediately post-injury**					
Rested from academics only		41	2.9		
Rested from exertion only		334	23.8		
Rested from both		953	67.9		
Rested from neither		76	5.4		
Days to full academics by initial rest profile					
Rested from academics only				5	3–13
Rested from exertion only				1	0–2
Rested from both				9	5–18
Rested from neither				2	1–2
Days to exertion by initial rest profile					
Rested from academics only				2	2–2
Rested from exertion only				7	4–11
Rested from both				11	6–21
Rested from neither				2	2–2
**Return-to-activity stages**					
Days post-injury when athlete reached five stages					
Symptom resolution				8	4–17
Academics				6	2–13
Exertion				9	5–16
Limited play				11	7–21
Full play				14	9–25
Returned through return-to-sport protocol sequence as expected		911	84.5		
Received academic accommodations		513	47.1		
**Recovery outcomes**					
Days to symptom resolution				8	4–17
Return to full play ≤14 days post-injury		753	52.2		
Return to full play ≤21 days post-injury		1006	69.8		
Prolonged return to full play >28 days post-injury		305	21.2		

### Days to symptom resolution

In athletes who had symptom resolution ≤14 days post-injury, the incidence of symptom resolution was most common on day 2 post-injury and decreased steadily as days elapsed post-injury (online supplemental figure 1).

10.1136/bjsports-2021-104451.supp1 Supplementary data

### Profiles describing sequence of return

**Figure 1:**
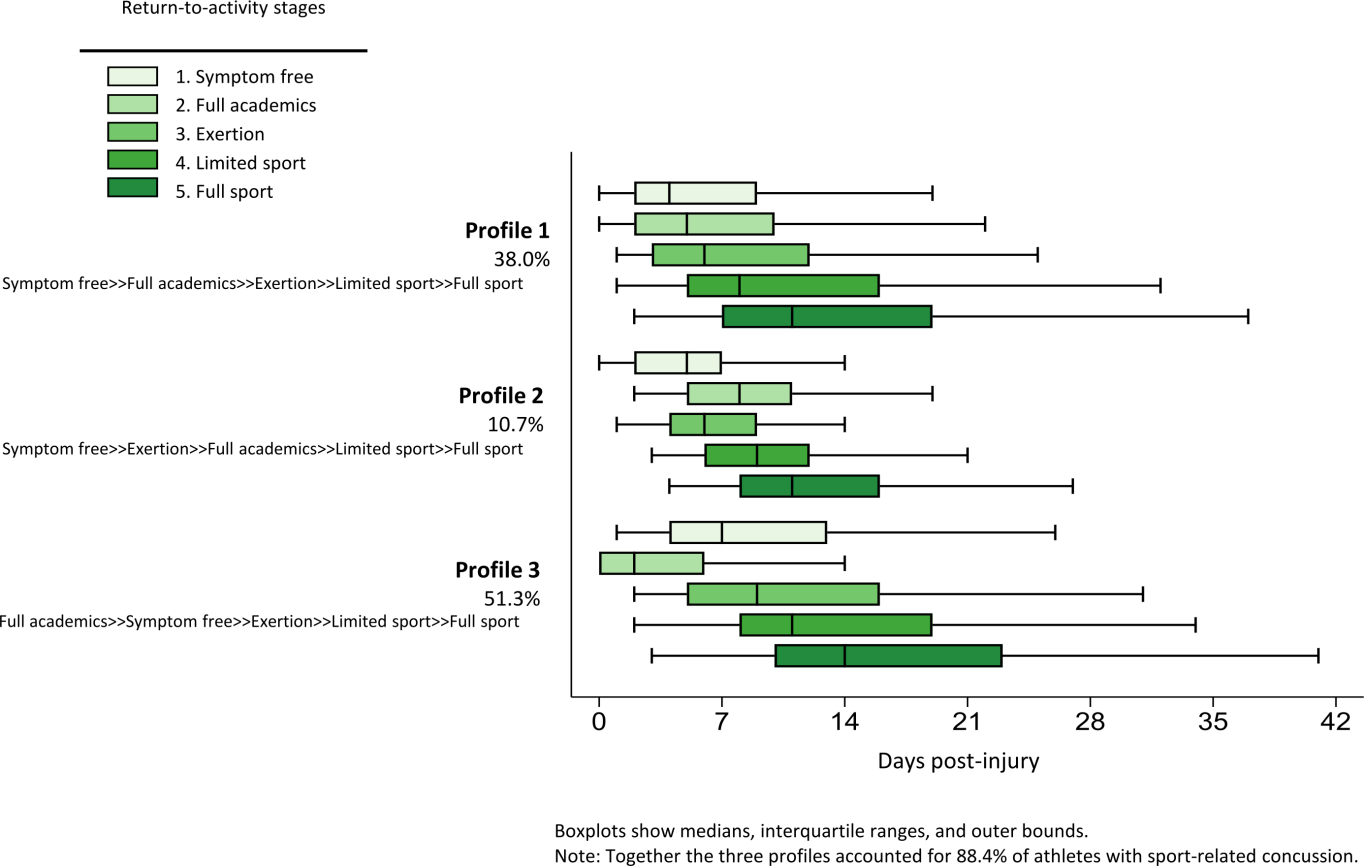
Three recovery profiles defined the sequence in which athletes reached stages relevant to return-to-activity stages after sport-related concussion. Profiles varied in timing of return to full academics, yet all adhered to sport-specific Consensus in Sport Group guidelines.

Figure 1 shows that three recovery profiles accounted for how the majority (88.4%) of athletes reached the stages. Approximately one-third (38.0%) had symptom resolution first, and then returned to full academics, physical exertion, limited sport and full sport, respectively (Profile 1); which is most consistent with the 2017 CISG consensus recommendations. In Profile 2, 10.7% had symptom resolution first, followed by initiating physical exertion, then return to full academics, then limited sport, then full sport. The most common sequence, exhibited by 51.3% of athletes, was return to full academics first, then symptom resolution, then physical exertion, limited sport and full sport (Profile 3).

The profiles did not differ significantly in the percent of athletes with ≥1 previous concussion (**χ** ^2^=1.52, p=0.47), proportion female (**χ** ^2^=3.42, p=0.18) nor percent from each athletic conference (**χ** ^2^=2.38, p=0.31). The profiles did significantly differ in having an initial 24–48 hour period of rest, however, which was exhibited by 75.0% of athletes in Profile 1 and 83.3% of athletes in Profile 2, but 47.8% of athletes in Profile 3 (**χ** ^2^=70.64, p<0.01). The median symptom count was 8 (IQR=5–12) in Profile 1, 9 (IQR=5–12) in Profile 2 and 9 (IQR=6–14, **χ** ^2^=5.11, p=0.08) in Profile 3.

An additional 16 sequences were exhibited in the remaining 6.6% of athletes; each was uncommon, accounting for <2% of athletes. In the sample overall, very few (5.0%) athletes initiated physical exertion as a first event.

A similar proportion of athletes followed Profile 3 before (52.2%) and after (49.6%) the 2017 CISG consensus statement. The prevalence of Profile 1, where full academics occurred before exertion activities, decreased from 40.5% to 33.3% after the 2017 statement, and the prevalence of Profile 2 where exertion occurred before full academics increased from 7.3% to 17.1% (p<0.001).

#### Recovery profile and days to full sport

**Figure 2:**
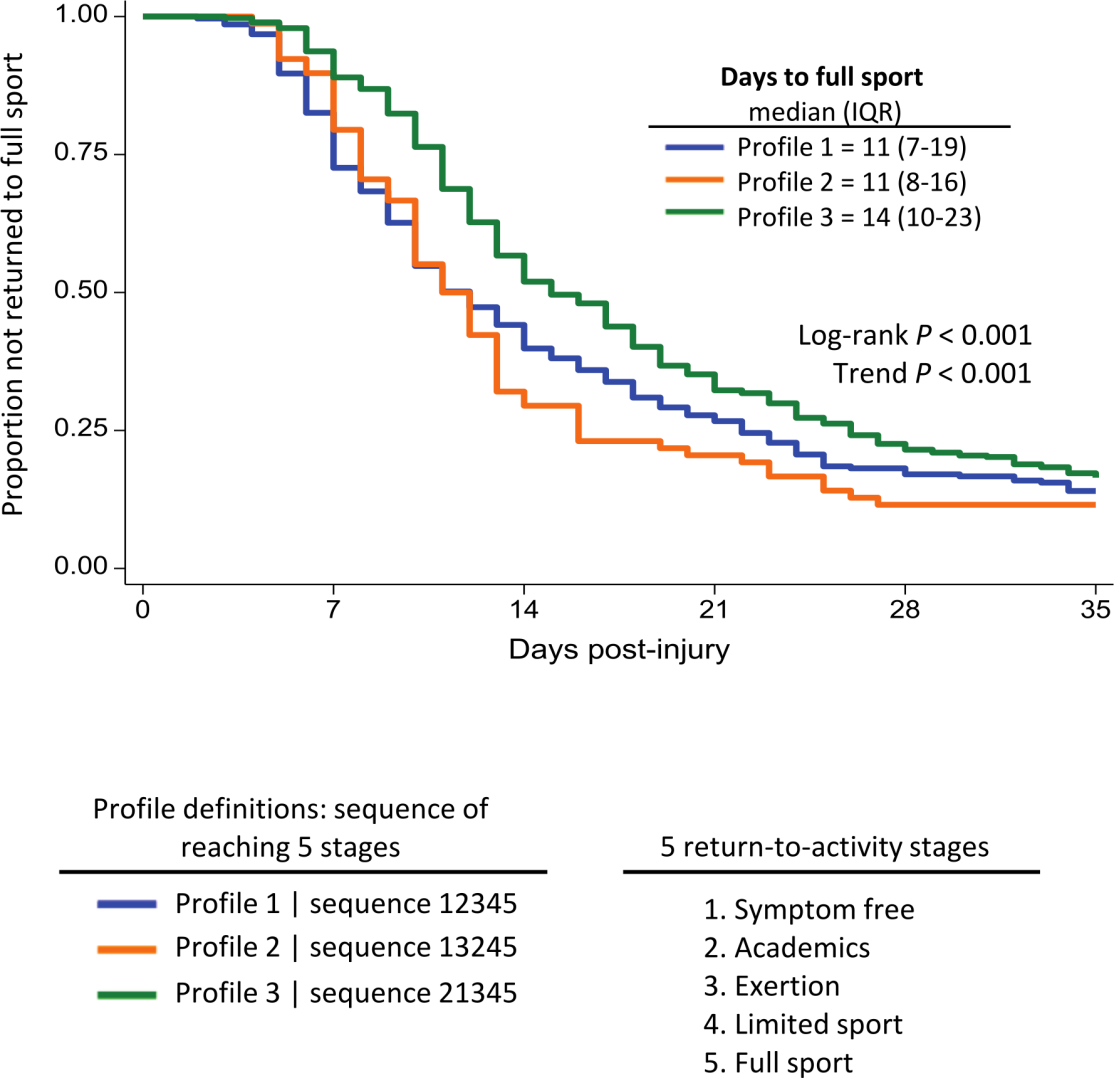
Unadjusted Kaplan-Meier curves displaying the median time to return to full sport after sport-related concussion was longest in Profile 3 (14 days), in which athletes initiated full academics first relative to other stages, compared with Profiles 1 and 2 (p<0.001).

Figure 2 shows the unadjusted Kaplan-Meier analysis of days to return to full sport for athletes in each of the three profiles. The median time to full sport was 11 days for athletes in the two profiles where symptom resolution occurred as the first stage (Profile 1 and Profile 2), and was significantly longer (14 days, p<0.001) in Profile 3 where athletes returned to full academics first.

### Timing of recovery stages relative to symptom resolution

**Figure 3:**
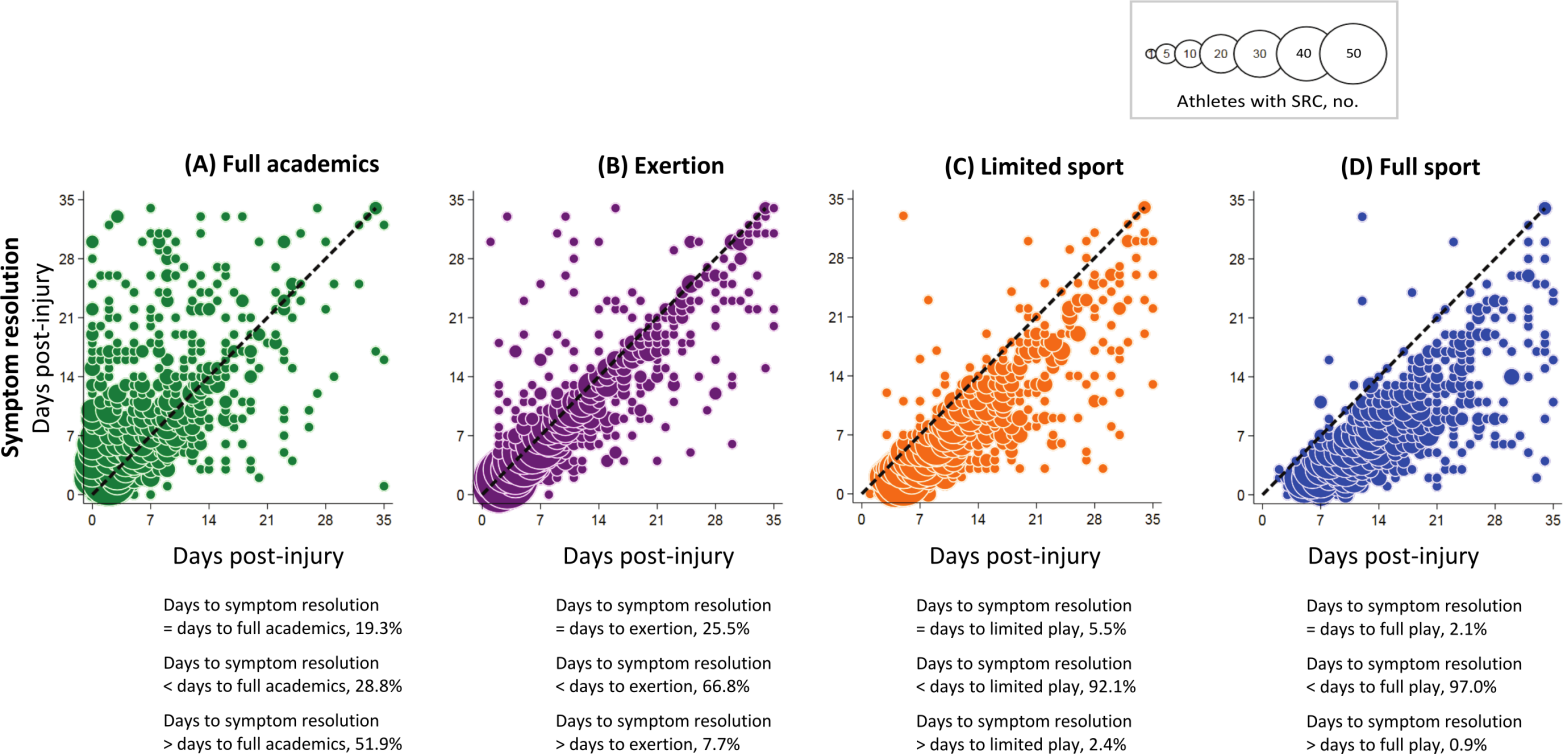
Scatterplots showing the number of athletes with sport-related concussion by the timing of symptom resolution post-injury relative to the timing of return to activity stage. (A) Half (51.9%) returned to full academics before symptom resolution, whereas (B) 66.0% returned to exertion, (C) 92.1% returned to limited sport and (D) 97.0% returned to full sport after symptom resolution.

Figure 3 shows scatterplots of the timing when (ie, days post-injury) each athlete reached each of the stages relative to when they experienced symptom resolution. One quarter (25.5%) initiated physical exertion on the same day symptoms resolved, two-thirds (66.8%) initiated exertion only after symptoms resolved, and few (7.7%) initiated exertion before symptoms resolved (figure 3B). In contrast, 19.3% of athletes returned to full academics on the day symptoms resolved, one quarter (28.8%) returned to full academics only after symptoms resolved, whereas half the athletes (51.9%) returned to full academics before symptoms resolved (figure 3A). The great majority of athletes initiated limited sport (figure 3C) and full sport (figure 3D) only after symptoms resolved.

#### Days to full sport based on timing of exertion and full academics relative to symptom resolution

**Figure 4:**
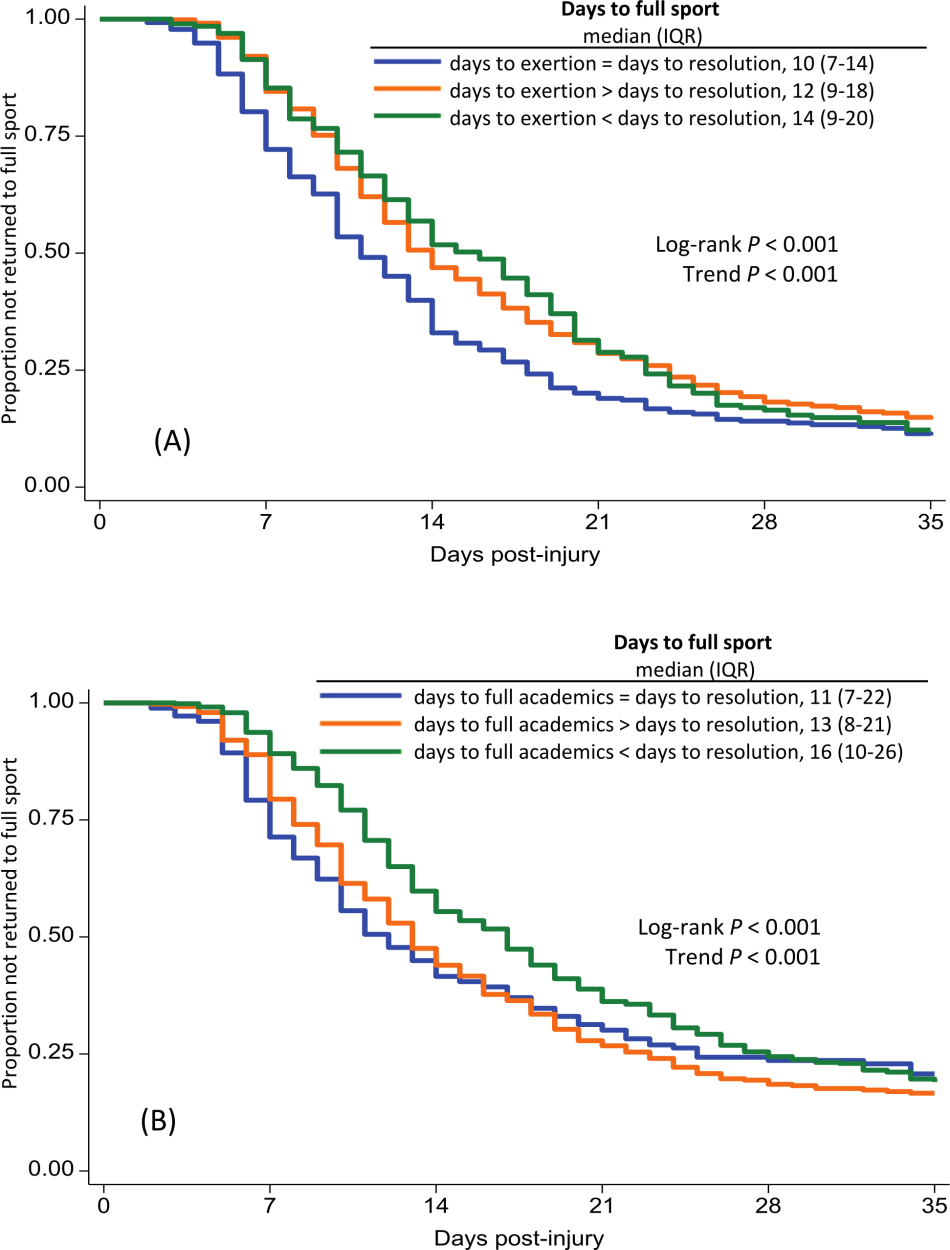
Unadjusted Kaplan-Meier curves showing the median time to return to full sport among athletes with sport-related concussion was longest if they (A) started exertion activities before symptoms resolved (p<0.001) or (B) returned to full academics before symptoms resolved (p<0.001).

The unadjusted Kaplan-Meier results in figure 4A show that days to return to full sport was shortest in athletes who returned to exertion on the same day their symptoms resolved (median=10 days), 2 days longer (12 days) in athletes who initiated exertion after symptoms resolved and 4 days longer (14 days, p<0.001) in athletes who initiated exertion before symptoms resolved (figure 4A). Analogously, figure 4B shows that days to full sport was shortest in athletes who returned to academics on the same day symptoms resolved (11 days), and longest for athletes who returned to academics before symptoms resolved (16 days, p<0.001).

### Multivariable logistic regression to identify risk factors of return to full sport ≤14 days

**Figure 5:**
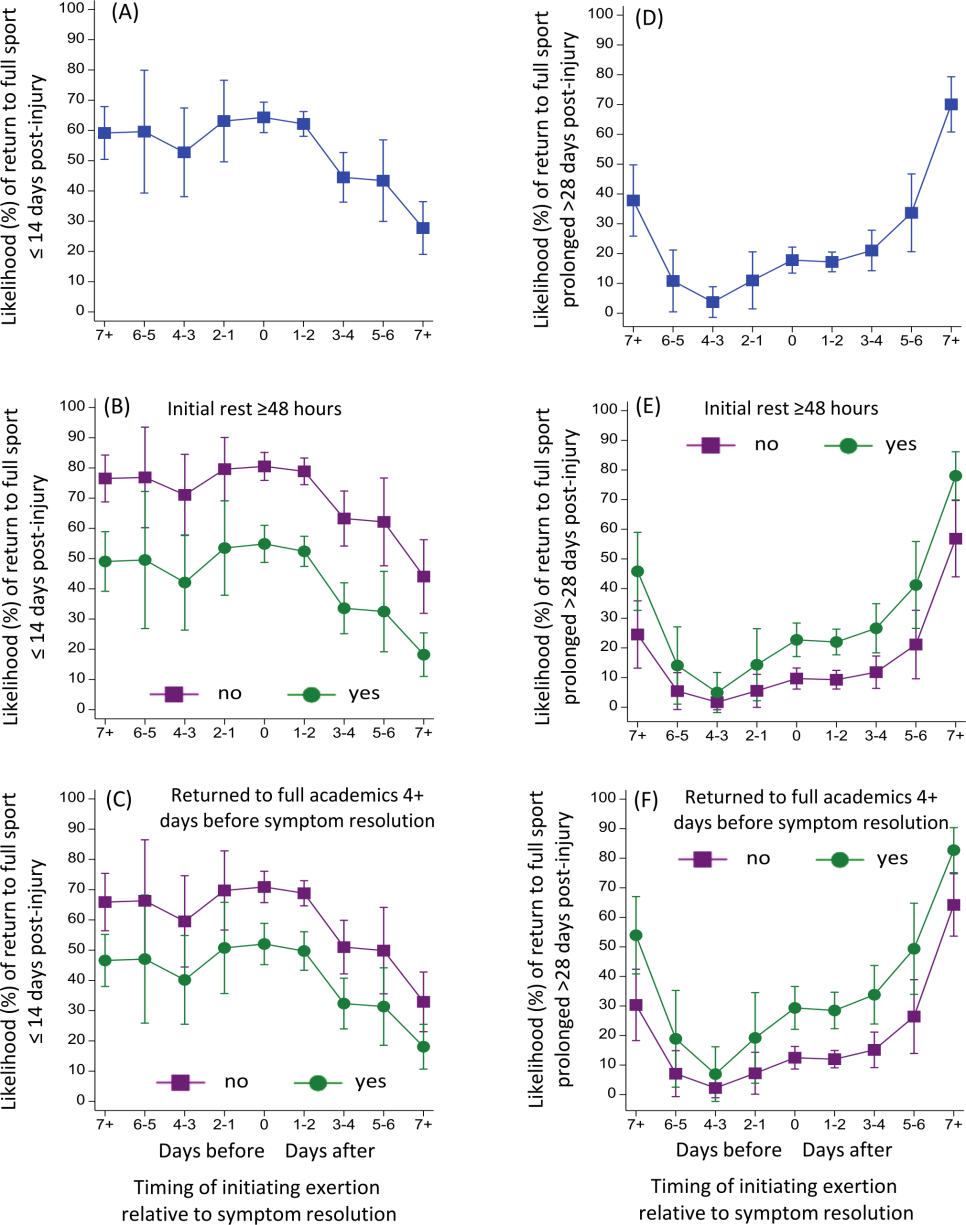
The predicted likelihood of returning to full sport ≤14 days (A) was progressively lower with more days elapsing between initiating exertion activities after symptom resolution occurred. Additionally, the likelihood of returning to full sport ≤14 days was systematically lower in athletes that (B) had initial 24–48 hours rest and (C) initiated academics 4+ days before symptom resolution. The predicted likelihood of returning to full sport >28 days (D) was progressively higher with more days elapsing between initiating exertion activities after symptom resolution occurred. Additionally, the likelihood of returning to full sport >28 days was systematically higher in athletes that (E) had initial 24–48 hours rest and (F) initiated academics 4+ days before symptom resolution.

Having 24–48 hours initial physical and cognitive rest was associated with lower likelihood of return to full sport ≤14 days (ARD −25.1%, 95% CI −30.7% to −19.5%) (table 2D). While controlling for 24–48 hours initial rest and the additional potential confounders, the likelihood of returning to full sport ≤14 days was 64.3% in athletes who initiated exertion on the same day of symptom resolution (table 2A and figure 5A) and was not statistically different in athletes who initiated exertion before symptom resolution (table 2D and figure 5A). In contrast, in athletes who initiated exertion after symptom resolution, the likelihood of returning to full sport ≤14 days was progressively lower as days additional days elapsed between the two stages (table 2D and figure 5A).

**Table 2:** Likelihood of return to full sport ≤14 days or ≤21 days or return to full sport prolonged >28 days among athletes with SRC, and absolute risk differences based on having 48 hours initial rest and returning to exertion or full academics on the same day or before or after symptom resolution

RISKS	(A)			(B)			(C)		
	Return to full sport			Return to full sport			Prolonged return to full sport		
	≤14 days			≤21 days			>28 days		
	Likelihood	95% CI		Likelihood	95% CI		Likelihood	95% CI	
**Physical and cognitive rest 48 hours**									
Yes	47.9	44.6	51.3	74.4	71.4	77.5	27.8	24.9	30.7
No	73.1	68.9	77.2	86.5	83.2	89.7	14.8	11.5	18.2
**Exertion relative to symptom resolution, days**									
7+ before	59.2	50.5	67.9	69.4	54.8	84.0	37.0	25.2	48.8
5–6 before	59.2	38.7	79.6	91.2	81.1	101.3	10.7	0.5	20.9
3–4 before	53.3	38.7	67.8	87.6	78.5	96.7	3.6	−1.4	8.6
1–2 before	62.7	49.1	76.3	87.8	78.2	97.3	11.5	1.6	21.4
0	64.3	59.3	69.4	88.8	85.1	92.6	17.7	13.4	22.0
1–2 after	62.0	58.0	66.1	82.6	79.2	86.0	17.5	14.1	20.8
3–4 after	44.4	36.2	52.7	69.7	61.8	77.7	21.3	14.5	28.1
5–6 after	44.1	30.6	57.6	70.9	57.6	84.2	33.1	20.2	45.9
7+ after	28.0	19.2	36.9	39.6	29.2	50.0	69.4	60.0	78.7
**Academics 4+ days before symptom resolution**									
Yes	42.1	37.2	46.9	62.7	57.0	68.3	37.1	32.1	42,2
No	63.5	60.5	66.5	84.1	81.8	86.4	18.1	15.8	20.3

	(D)			(E)			(F)		
RISK DIFFERENCES	ARD	95% CI		ARD	95% CI		ARD	95% CI	
**Physical and cognitive rest 48 hours**									
Y vs N	−25.1	−30.7	−19.5*	−12.0	−16.8	−7.3*	12.9	8.3	17.6*
**Exertion relative to symptom resolution, days**									
7+ before vs day 0	−5.2	−15.3	5.0	−19.4	−34.4	−4.3*	19.3	6.7	31.9*
5–6 before vs day 0	−5.2	−26.2	15.9	2.4	−8.4	13.1	−7.0	−18.1	4.1
3–4 before vs day 0	−11.1	−26.5	4.4	−1.2	−11.0	8.7	−14.1	−20.7	−7.5*
1–2 before vs day 0	−1.7	−16.1	12.8	−1.1	−11.3	9.2	−6.2	−16.9	4.5
1–2 after vs day 0	−2.3	−8.8	4.1*	−6.2	−11.3	−1.2*	−0.3	−5.7	5.2
3–4 after vs day 0	−19.9	−29.6	−10.2*	−19.1	−27.9	−10.3*	3.6	−4.4	11.6
5–6 after vs day 0	−20.3	−34.7	−5.8*	−17.9	−31.8	−4.0*	15.4	1.8	28.9*
7+ after vs day 0	−36.3	−46.5	−26.1*	−49.2	−60.4	−38.1*	51.6	41.2	62.0*
**Early academics**									
Y vs N	−21.5	−27.4	−15.5*	−21.4	−27.7	−15.2*	19.1	13.4	24.7

Estimated with logistic regression adjusting for athlete sex, number of symptoms, number of previous concussions, and returning to full academics before symptom resolution. Early academics defined as initiating academic activities 4+ days before symptom resolution.

*P<0.05 for absoluted risk differences.

ARD, absolute risk difference; SRC, sport-related concussion.

Modelling the academic stage of the return sequence revealed 4+ days prior to symptom resolution as a relative threshold for early academic return. Specifically, initiating academics 4+ days before symptom resolution was associated with a 21.5% lower likelihood (95% CI −27.4% to −15.5%) of returning to full sport ≤14 days (table 2D) after controlling for initial rest, timing of exertion relative to symptom resolution and the additional confounders.

Our tests for effect modification identified that athletes who had initial 24–48 hours physical and cognitive rest were less likely to return to full sport ≤14 days regardless of whether they initiated exertion before, on or after the day they experienced symptom resolution (online supplemental table 1 and figure 5B). Analogously, athletes who returned to academics 4+ days prior to symptom resolution were less likely to return to full sport ≤14 regardless of whether they initiated exertion before, on or after they day they experienced symptom resolution (online supplemental table 1 and figure 5C). Results were similar when using return to full sport ≤21 days as the outcome as a sensitivity analysis (table 2B and E, online supplemental table 1, online supplemental figure S2).

10.1136/bjsports-2021-104451.supp3 Supplementary data

10.1136/bjsports-2021-104451.supp2 Supplementary data

### Multivariable logistic regression to identify risk factors of prolonged return to full sport >28 days

Having 24–48 hours initial physical and cognitive rest was associated with a higher likelihood that return to full sport would be prolonged >28 days (ARD 12.9%, 95% CI 8.3% to 17.6%) (table 2F), and initiating academics 4+ days prior to symptom resolution was associated with a higher likelihood of prolonged delay to full sport (ARD 19.1%, 95% CI 13.4% to 24.7%) (table 2F). While taking these into account, the likelihood that athletes’ return to full sport would be prolonged >28 days was 17.7% (95% CI 13.4% to 22.0%) for those who returned to exertion on the same day as symptom resolution (table 2C and figure 5D). Compared with these athletes, initiating exertion considerably before (ie, 7+ days) symptom resolution was associated with a 19.3% higher likelihood (95% CI 6.7% to 31.9%) of prolonged delay to full sport (table 2F and figure 5D). In contrast, initiating exertion only 3–4 days before symptom resolution was associated with a 14.1% lower likelihood (95% CI −20.7% to −7.5%) of prolonged delay to full sport (table 2F and figure 5D).

Athletes who had initial 24–48 hours physical and cognitive rest were more likely to have prolonged return to full sport >28 days regardless of whether they initiated exertion before, on or after the day they experienced symptom resolution (online supplemental table 1 and figure 5E). Analogously, athletes who returned to academics 4+ days prior to symptom resolution were more likely to have prolonged return to full sport >28 regardless of whether they initiated exertion before, on or after they day they experienced symptom resolution (online supplemental table 1 and figure 5F).

## Discussion

Most athletes with SRC progressed through an RTS protocol as expected based on a determination from the athletes’ clinicians. Also, most athletes reached stages of the protocol in an order generally consistent with best practice guidelines that are relevant to the collegiate-athlete population; namely, the 2017 CISG consensus statement^3^ and 2014 NATA position statement.^1^ Those guidelines are specific regarding return-to-sport, but are comparatively vague regarding academic activities,^13^ which may explain why the timing of resuming full academics varied the most of the five stages we studied. We found that time to return to full play varied systematically based on stages of return relative to symptom resolution, specifically return to full academics and initiation of exertion. Two-thirds of the athletes exhibited a brief period of physical and cognitive rest (ie, 24–48 hours) during the acute phase of SRC, which we controlled for in the analysis. Taken together, the results suggest concussion management teams adhere to current guidelines, and the timing of each return stage corresponds to overall time to return to sport; however, future guidelines should address the timing of academic return more directly and specifically for collegiate athletes.^13^ To our knowledge, this study is the first to report on a large population of athletes with SRC and the timing of reaching stages relevant to a RTS protocol. Below we discuss the findings and contributions, including evidence in support of CISG and NATA recommendations and evidence that could inform updates.

### Brief initial physical and cognitive rest

Two of every three athletes (67.9%) had an initial 24–48 hours of physical and cognitive rest before starting an RTS progression. These athletes endorsed more symptoms than did others, and took longer for symptoms to resolve, suggesting that having 24–48 hours initial rest may correspond to SRC severity. Initial physical and cognitive rest became more common over the study period, consistent with the possibility that athletic trainers were adopting the recommendation for brief initial rest included in 2017 CISG consensus statement.

Note that initial physical and cognitive rest was associated with lower likelihood of return to full sport ≤14 days, and higher likelihood of prolonged full sport >28 days. This may be in part due to acute rest setting back the date when the athlete could begin a RTS progression. As such, this finding should not be interpreted to mean that rest is detrimental. Another factor is athletes’ symptom experiences, as those who completed 24–48 hours of initial rest endorsed more symptoms. This could explain why an initial 24–48 hours of rest was associated with a delay to symptom resolution and full sport, but cannot be understood definitively here given the observational nature of the study.

### Sequential progression through return stages

The sequential progression exhibited by student athletes with SRC indicates that athletic trainers are providing active management consistent with best practice recommendations of the CISG and NATA. This study did not set out to explicitly measure RTS stages as defined by either the CISG or NATA. Rather, the data include dates of key milestones that map to the stages defined in the CISG and NATA guidelines. Importantly, whereas the CISG consensus statement describes a return-to-academics strategy that is separate from their RTS (ie, return-to-sport) progression, we captured time (ie, days post-injury) to return to full academics. This was one of five return-to-activity stages we used to investigate their timing relative to each other, and their relation to the outcome of interest, return to full sport.

Using these five stages let us detect that the most common progression, exhibited by 51.3% of the athletes, involved returning to full academics first, then experiencing symptom resolution, followed by initiating exertion, limited sport and full sport (Profile 3). However, 38.0% of our sample exhibited Profile 1: symptom resolution, return to full academics, initiating exertion followed by a gradual re-introduction to sport. Profile 1, in general, most closely corresponds to the 2017 CISG consensus statement recommending symptom-limited activities, including a gradual re-introduction of school activities, that do not provoke symptoms early during the return-to-sport strategy. Importantly, we found that athletes in all three profiles adhered to CISG guidelines, in that sport-specific return stages occurred after symptom resolution; however, we identified variability as to when return-to-academics occurred in the sequential stages of return. Notably, unadjusted analyses identified that athletes within Profile 3, with academic return as their first stage, exhibited delayed RTS, as the median time to full sport was 14 days compared with 11 days in athletes in Profiles 1 and 2.

An early intervention in concussion management that is increasingly accepted includes sub-symptom threshold activity, which provides evidence in decreasing symptom burden,^14^ and additional research supporting this notion was published since the 2017 CISG consensus statement was reported.^15–18^ In the present study, we were not able to detect whether or when athletes may have pursued subthreshold exercise as treatment for concussion. Instead, we measured the initiation of exertion, where the median time was considerably after the injury date (ie, 9 days). Our profiles indicated that the majority of athletes began exertion activities after symptom resolution, and therefore, future research should further investigate sub-symptom threshold exercise in early stages of an RTS protocol.

### Timing of exertion initiation relative to symptom resolution influences days to full sport

The shortest time to return to full sport ≤14 days occurred in athletes who initiated sport-related exertion on the same day their symptoms resolved, which was a median 2 days longer in athletes who initiated exertion after symptoms resolved, and 4 days longer in athletes who initiated exertion before symptoms resolved. Further, initiating exertion just 3–4 days prior to symptom resolution was associated with decreased likelihood of a prolonged return to full sport >28 days. In contrast, initiating exertion considerably before (7+ days) symptom resolution corresponded to a higher likelihood of delayed recovery beyond 28 days. These are novel findings, suggesting there may be prolonged-delay consequences of returning an athlete to sport too quickly; yet, there may be benefits of starting exertion at an appropriate time shortly before symptom resolution.^18^ Often, an athlete’s progress on the timeline to symptom resolution is only known in retrospect, emphasising the importance of continual monitoring of athletes’ symptom recovery. This observational study could motivate future research using approaches including real-time monitoring^19^ to identify whether an optimal time to introduce exertional activity exists. In addition, those with prolonged recovery >28 days generally had a greater number of symptoms. We controlled for this as this may be a potential reason for the longer initial rest period or delay in initiation of a return protocol. Accordingly, the results of this study should motivate further research to identify appropriate timing and intensities for sport-related exertion activities as part of an RTS progression in collegiate athletes, and the benefits or consequences of initiating exertion activities relative to symptomology.

### Timing of full academics relative to symptom resolution influences days to full sport

Initiating full academics 4+ days before symptom resolution was associated with lower likelihood of return to full sport ≤14 days, and higher likelihood of prolonged recovery >28 days. This is consistent with studies finding that athletes who return to academics before their symptoms resolve may be overexerting themselves cognitively or prioritising academics results in delayed recovery, but is the first evidence from a large collegiate-athlete population. In a study of patients presenting to a sports concussion clinic, higher self-reported cognitive activity was associated with a longer duration of symptoms.^20^ That study included patients who presented after a considerable delay (ie, up to 3 weeks), however, and the median age was 15 years and a large percentage of patients (19%) had loss of consciousness. Moreover, a chart review found that almost half (44.7%) of the student athletes treated at a sport medicine practice returned to school too soon, as evidenced by a relapse or recurrence of symptoms.^21^ Only 20% of those students were in college, however, with the remainder in elementary and high school. Taken together, this is evidence that consensus guidelines should consider having cognitive or academic activities explicitly in an RTS protocol. Also, the CISG and NATA should consider updating their guidance related to academics to include students participating at the collegiate level.

### Clinical implications

The sequence by which athletes complete stages of an RTS protocol may be the result of many factors in addition to oversight by an athletic trainer and their integration with consensus guidelines,^1^ including rationale of different guidelines^22^ as well as pressure and priorities of key stakeholders involved in concussion management.^23^ For example, in a survey of college athletic trainers, 36% indicated they have not felt pressured to return an athlete to the classroom after a concussion, whereas only 18% reported no pressure to return an athlete to physical activity.^23^ Another factor may be student athletes’ or clinicians’ priorities for returning collegiate athletes to sport prior to or after full classroom participation without restrictions, as previous research identified 13% of athletic trainers were neutral and 77% agreed that classroom participation must come first.^23^

In the collegiate setting, members of the interdisciplinary concussion management team must adhere to the NCAA concussion management checklist.^7^ Therefore, although our results suggest some variation in return to activity progressions, mandated homogeneity in concussion management exists at this level. The differences may exist as result of the ability of, and way in which, sites carry out guidelines, involvement or inclusion of members of the multidisciplinary care team, variability in resources, and individuality in concussion management. Importantly, the findings in the current study demonstrate the utility of individualised concussion management plans,^24 25^ as clinical impairments and needs of student-athletes following SRC are not uniform. Accordingly, this study should motivate future work to identify particular reasons for the different progression profiles that we identified in this collegiate student athlete population.

### Limitations

The results may not be generalisable to non-contact or non-collision sport athletes, or athletes participating in levels of competition other than college. Also, we defined each athletes’ experiences after SRC based on dates when they reached a relevant outcome, rather than a more nuanced accounting of levels of exertion, both physical and cognitive. In addition, the data lack a number of comorbid factors that may influence recovery after SRC, including migraine, psychiatric condition, or learning disability. Further, it is unknown whether athletes or members of their concussion management team prioritised one outcome over another (eg, academics vs sport). Future work could prospectively monitor physical and cognitive activity that athletes exert through a RTS progression, and determine whether duration and intensity affects recovery. Also, while we were able to control for symptom burden as measured by symptom count, we were not able to control for symptom severity which has been suggested be a consistent predictor of delayed recovery outcomes.^23 26^ The CISG^3^ and NATA^1^ both recommend academic accommodations as part of a return-to-school strategy. Our data include only a yes/no indicator of whether a student athlete received academic accommodations, which prevented us from studying their role in recovery. Finally, we cannot establish whether an athlete’s condition due to SRC affected when they reached each outcome, versus whether their progression through RTS stages affected their time to recovery given the observational nature of the study. Being a prospective cohort study, however, along with other study design features that are strong including a large sample and detailed, date-specific data from many sites, do elevate the strength of our evidence that the timing of early or late exertion and full academics are associated with prognosis in terms of time to full sport participation.

## Conclusions

Collegiate student athletes exhibited adherence to current guidelines for a graduated RTS protocol after SRC. The pace at which outcomes were reached was associated with when exertion and academic activities were initiated, relative to when an athlete’s symptoms resolved. While controlling for potential confounders, we observed the shortest return to full sport in athletes who initiated sport-related exertion on the same day their symptoms resolved; while initiating exertion considerably early (+7 days) relative to symptom resolution increased the likelihood of prolonged return to full sport (>28 days), and initiating exertion 3–4 days prior to symptom resolution decreased the likelihood of a prolonged return. These findings reveal the importance to simultaneously consider how initial rest, physical exertion and academic activities interact, given our evidence that the time to return to full sport was a function of when these components of a return-to-sport protocol occur relative to each other.

**Key messages**

**What is already known about this topic**

- Consensus guidelines recommend a stepwise progression for sport-related concussion (SRC) management.
- The consensus guidelines are based largely on expert opinion, and whether they are followed in the collegiate setting and how they affect recovery is not known.

**What this study adds**

- We established that there is adherence to sport-specific return-to-activity recommendations, providing support that consensus guidelines direct clinical practice.
- The timing of stages relative to one another relates to return to full sport, and academic activity was an important correlate of SRC recovery in collegiate student athletes.

**How this might affect research, practice or policy**

- The results provide an evidence base for the consensus guidelines and also reveal a need to better understand and clarify the recommended timing of academics as an early stage of SRC management.

## Data Availability

No data are available. Not applicable.

## References

[1] Broglio SP , Cantu RC , Gioia GA , et al . National Athletic Trainers’ Association Position Statement: Management of Sport Concussion. J Athl Train 2014;49:245–65. 10.4085/1062-6050-49.1.07 24601910 PMC3975780

[2] Lempke LB , Schmidt JD , Lynall RC . Athletic trainers' Concussion-Assessment and Concussion-Management practices: an update. J Athl Train 2020;55:17–26. 10.4085/1062-6050-322-18 31855075 PMC6961637

[3] McCrory P , Meeuwisse W , Dvořák J , et al . Consensus statement on concussion in sport-the 5^th^ international conference on concussion in sport held in Berlin, October 2016. Br J Sports Med 2017;51:838–47. 10.1136/bjsports-2017-097699 28446457

[4] McCrory P , Meeuwisse WH , Aubry M , et al . Consensus statement on concussion in sport: the 4th International Conference on concussion in sport held in Zurich, November 2012. Br J Sports Med 2013;47:250–8. 10.1136/bjsports-2013-092313 23479479

[5] American Academy of Pediatrics . Recommendations for participation in competitive sports. Pediatrics 1988;81:737. 3357741

[6] Sport concussion assessment tool - 5th edition. British Journal of Sports Medicine 2017;51:851. 28446451 10.1136/bjsports-2017-097506SCAT5

[7] NCAA . Concussion safety protocol checklist, 2021. Available: https://ncaaorg.s3.amazonaws.com/ssi/concussion/2021_Concussion_Safety_Protocol_Checklist.pdf

[8] Putukian M , D’Alonzo BA , Campbell-McGovern CS , et al . The ivy League–Big ten epidemiology of concussion study: a report on methods and first findings. Am J Sports Med 2019;47:1236–47. 10.1177/0363546519830100 30943078

[9] Hosmer DW , Hosmer T , Le Cessie S , et al . A comparison of goodness-of-fit tests for the logistic regression model. Stat Med 1997;16:965–80. 10.1002/(SICI)1097-0258(19970515)16:9&lt;965::AID-SIM509&gt;3.0.CO;2-O 9160492

[10] Hosmer DW , Taber S , Lemeshow S . The importance of assessing the fit of logistic regression models: a case study. Am J Public Health 1991;81:1630–5. 10.2105/AJPH.81.12.1630 1746660 PMC1405276

[11] Sterne JAC , White IR , Carlin JB , et al . Multiple imputation for missing data in epidemiological and clinical research: potential and pitfalls.. In: Bmj (clinical research. 338, 2009: b2393. 10.1136/bmj.b2393 PMC2714692 19564179

[12] Madley-Dowd P , Hughes R , Tilling K , et al . The proportion of missing data should not be used to guide decisions on multiple imputation. J Clin Epidemiol 2019;110:63–73. 10.1016/j.jclinepi.2019.02.016 30878639 PMC6547017

[13] Buckley TA , Baugh CM , Meehan WP , et al . Concussion management plan compliance: a study of NCAA power 5 conference schools. Orthop J Sports Med 2017;5:232596711770260. 10.1177/2325967117702606 PMC5407527 28473995

[14] Reid SA , Farbenblum J , McLeod S . Do physical interventions improve outcomes following concussion: a systematic review and meta-analysis? Br J Sports Med 2022;56:292–8. 10.1136/bjsports-2020-103470 34593371

[15] Langevin P , FRÉMONT P , Fait P , et al . Aerobic exercise for sport-related concussion: a systematic review and meta-analysis. Medicine Science in Sports Exercise 2020;52:2491–9. 10.1249/MSS.0000000000002402 32520867

[16] Powell C , McCaulley B , Scott Brosky Z , et al . The effect of aerobic exercise on adolescent athletes post concussion: a systematic review and meta-analysis. Int J Sports Phys Ther 2020;15:650–8. 10.26603/ijspt20200650 33110684 PMC7566833

[17] Ledoux A-A , Barrowman NJ , Boutis K , et al . Multicentre, randomised clinical trial of paediatric concussion assessment of rest and exertion (PedCARE): a study to determine when to resume physical activities following concussion in children. Br J Sports Med 2019;53:195. 10.1136/bjsports-2017-097981 28701360

[18] Leddy JJ , Master CL , Mannix R , et al . Early targeted heart rate aerobic exercise versus placebo stretching for sport-related concussion in adolescents: a randomised controlled trial. The Lancet Child Adolesc Health 2021;5:792–9. 10.1016/S2352-4642(21)00267-4 34600629

[19] Wiebe DJ , Nance ML , Houseknecht E , et al . Ecologic momentary assessment to accomplish real-time capture of symptom progression and the physical and cognitive activities of patients daily following concussion. JAMA Pediatr 2016;170:1108–10. 10.1001/jamapediatrics.2016.1979 27617669

[20] Brown NJ , Mannix RC , O’Brien MJ , et al . Effect of cognitive activity level on duration of post-concussion symptoms. Pediatrics 2014;133:e299–304. 10.1542/peds.2013-2125 24394679 PMC3904277

[21] Carson JD , Lawrence DW , Kraft SA . Premature return to play and return to learn after a sport-related concussion: physician’s chart review. Can Fam Physician 2014;60:e310, e2-5. 24925965 PMC4055342

[22] Lempke LB , Schmidt JD , Lynall RC , et al . And Concussion-Management practices: an update. J Athl Train 2020;55:17–26. 31855075 10.4085/1062-6050-322-18 PMC6961637

[23] Williamson CL , Norte GE , Broshek DK , et al . Return to learn after sport-related concussion: a survey of secondary school and collegiate athletic trainers. J Athl Train 2018;53:990–1003. 10.4085/1062-6050-234-17 30398928 PMC6263072

[24] Sufrinko AM , Kontos AP , Apps JN , et al . The effectiveness of prescribed rest depends on initial presentation after concussion. J Pediatr 2017;185:167–72. 10.1016/j.jpeds.2017.02.072 28365025

[25] Ellis MJ , Leddy J , Cordingley D , et al . A physiological approach to assessment and rehabilitation of acute concussion in collegiate and professional athletes. Front Neurol 2018;9:1115. 10.3389/fneur.2018.01115 30619068 PMC6306465

[26] Iverson GL , Gardner AJ , Terry DP , et al . Predictors of clinical recovery from concussion: a systematic review. Br J Sports Med 2017;51:941–8. 10.1136/bjsports-2017-097729 28566342 PMC5466929

