# Supplementary material for: Progression through return-to-sport and return-to-academics guidelines for concussion management and recovery in collegiate student athletes: findings from the Ivy League–Big Ten Epidemiology of Concussion Study
Source: Br J Sports Med. 2022 Apr 20;56(14):801–11. doi: 10.1136/bjsports-2021-104451 (PMC9252856; doi:10.1136/bjsports-2021-104451)
Supplement: Supplementary data [file bjsports-2021-104451supp003.pdf]

Table Supp1. Likelihood of primary outcome and secondary outcomes among athletes with sport-related concussion, based on having 24-48 hours initial physical and cognitive rest and returning to full academics before symptom resolution.

	INITIAL 24-48 HOURS PHYS. AND COG. REST										FULL ACADEMICS 4+ DAYS BEFORE SYMPTOM RESOLUTION									
**(A) Return to full sport ≤14 days**[†]	YES			NO			YES - NO				YES			NO			YES - NO			
	LHD	95% CI		LHD	95% CI		ARD	95% CI			LHD	95% CI		LHD	95% CI		ARD	95% CI		
7+ before	48.9	38.9	58.8	76.1	68.2	84.1	-30.8	-37.7	-24.0	*	46.5	37.8	55.2	65.5	56.0	75.1	-22.7	-29.3	-16.1	*
5-6 before	49.3	26.5	72.1	76.4	59.5	93.4	-29.9	-36.4	-23.5	*	46.9	25.5	68.3	65.9	45.6	86.2	-22.5	-29.2	-15.9	*
3-4 before	42.4	26.8	58.1	71.1	57.7	84.5	-30.1	-36.6	-23.5	*	40.6	25.9	55.2	59.6	44.7	74.6	-21.9	-28.2	-15.5	*
1-2 before	53.9	38.2	69.6	79.6	69.1	90.2	-25.7	-33.8	-17.6	*	51.3	36.2	66.4	69.9	56.7	83.1	-20.1	-26.9	-13.2	*
0	55.1	48.9	61.3	80.4	75.7	85.1	-25.7	-31.4	-19.9	*	52.4	45.5	59.3	70.9	65.7	76.1	-20.9	-27.2	-14.7	*
1-2 after	52.4	47.4	57.5	78.7	74.2	83.1	-25.5	-30.9	-20.0	*	49.9	43.5	56.2	68.6	64.5	72.8	-21.2	-27.5	-14.8	*
3-4 after	33.6	25.1	42.1	63.0	53.8	72.2	-30.1	-36.6	-23.5	*	32.5	24.0	40.9	50.8	41.9	59.7	-21.6	-27.7	-15.5	*
5-6 after	33.0	19.7	46.3	62.4	47.7	77.0	-29.8	-36.2	-23.4	*	31.9	18.9	44.9	50.2	35.9	64.5	-21.4	-28.0	-14.7	*
7+ after	18.3	11.1	25.5	43.8	31.6	56.1	-25.0	-33.0	-17.0	*	18.2	10.7	25.6	32.8	22.9	42.7	-13.9	-19.1	-8.6	*
**(B) Return to full sport ≤21 days**[‡]	LHD	95% CI		LHD	95% CI		ARD	95% CI			LHD	95% CI		LHD	95% CI		ARD	95% CI		
7+ before	61.7	44.8	78.6	77.8	64.9	90.8	-23.8	-32.7	-15.0	*	51.3	33.3	69.3	74.9	60.6	89.2	-32.9	-41.5	-24.4	*
5-6 before	88.9	76.6	101	94.8	88.1	101	-10.0	-21.3	1.2		84.1	67.2	101	94.1	86.7	102	-15.4	-32.3	1.6	
3-4 before	84.2	72.8	95.5	92.2	85.6	98.8	-14.0	-22.8	-5.2	*	77.7	62.8	92.6	91.3	83.9	98.6	-21.4	-32.8	-10.0	*
1-2 before	85.4	74.1	96.8	92.9	86.6	99.2	-9.7	-17.5	-1.9	*	79.4	64.2	94.6	92.0	85.0	99.1	-16.6	-28.8	-4.5	*
0	86.0	81.2	90.8	93.2	90.3	96.1	-10.1	-14.6	-5.6	*	80.1	73.3	87.0	92.4	89.4	95.4	-17.3	-24.0	-10.6	*
1-2 after	78.7	74.3	83.0	89.0	85.5	92.5	-12.1	-16.6	-7.7	*	70.7	64.0	77.5	87.7	84.6	90.7	-22.9	-30.3	-15.5	*
3-4 after	63.4	54.0	72.8	79.1	71.5	86.6	-21.2	-28.9	-13.5	*	53.2	42.1	64.2	76.3	68.6	84.0	-31.8	-40.0	-23.7	*
5-6 after	64.4	49.0	79.7	79.7	68.0	91.5	-18.6	-26.7	-10.5	*	54.2	36.9	71.5	77.0	64.5	89.6	-32.1	-41.6	-22.5	*
7+ after	30.9	20.9	40.8	50.8	38.0	63.6	-24.3	-33.9	-14.6	*	22.6	13.2	32.0	45.3	33.9	56.7	-25.8	-33.5	-18.1	*
**(C) Prolonged return to full sport >28 days**[†]	LHD	95% CI		LHD	95% CI		ARD	95% CI			LHD	95% CI		LHD	95% CI		ARD	95% CI		
7+ before	45.8	32.5	59.0	24.4	13.1	35.8	25.2	16.7	33.7	*	54.1	41.0	67.2	30.3	18.2	42.4	28.0	20.4	35.6	*
5-6 before	14.1	1.1	27.2	5.4	-0.7	11.6	13.4	3.4	23.4	*	19.1	2.6	35.6	7.1	-0.6	14.9	16.5	4.8	28.1	*
3-4 before	4.9	-1.8	11.6	1.7	-0.8	4.2	4.4	-1.2	10.0		6.9	-2.3	16.2	2.2	-1.0	5.5	5.6	-1.5	12.8	
1-2 before	14.1	2.1	26.2	5.4	-0.1	10.9	8.6	1.2	15.9	*	19.1	3.8	34.4	7.1	0.1	14.1	10.5	1.8	19.2	*
0	22.7	17.1	28.3	9.6	6.1	13.1	13.3	8.2	18.4	*	29.5	22.3	36.6	12.4	8.6	16.2	17.2	11.4	23.1	*
1-2 after	22.0	17.7	26.3	9.3	6.1	12.4	11.8	7.6	15.9	*	28.7	22.5	34.8	12.0	9.1	14.9	17.0	11.2	22.8	*
3-4 after	26.8	18.5	35.1	11.8	6.4	17.2	15.8	9.5	22.2	*	34.1	24.2	44.0	15.2	9.2	21.2	20.5	13.3	27.7	*
5-6 after	41.4	26.8	55.9	21.2	9.6	32.7	20.6	12.7	28.5	*	49.7	34.3	65.2	26.5	14.1	38.9	26.8	18.8	34.7	*
7+ after	77.9	69.7	86.1	56.6	43.7	69.4	20.5	11.7	29.3	*	82.8	75.2	90.3	64.0	53.4	74.6	17.4	11.3	23.6	*

Values are graphed in †Figure 5 and ‡Figure Supp2.

LHD denotes likelihood. ARD denotes absolute risk difference.

* P < 0.05 for ARD values.

Estimated with logistic regression adjusting for athlete sex, number of symptoms, number of previous concussions, and returning to full academics before symptom resolution.
