# Supplementary material for: Progression through return-to-sport and return-to-academics guidelines for concussion management and recovery in collegiate student athletes: findings from the Ivy League–Big Ten Epidemiology of Concussion Study
Source: Br J Sports Med. 2022 Apr 20;56(14):801–11. doi: 10.1136/bjsports-2021-104451 (PMC9252856; doi:10.1136/bjsports-2021-104451)
Supplement: Supplementary data [file bjsports-2021-104451supp002.pdf]

Figure Supp2.

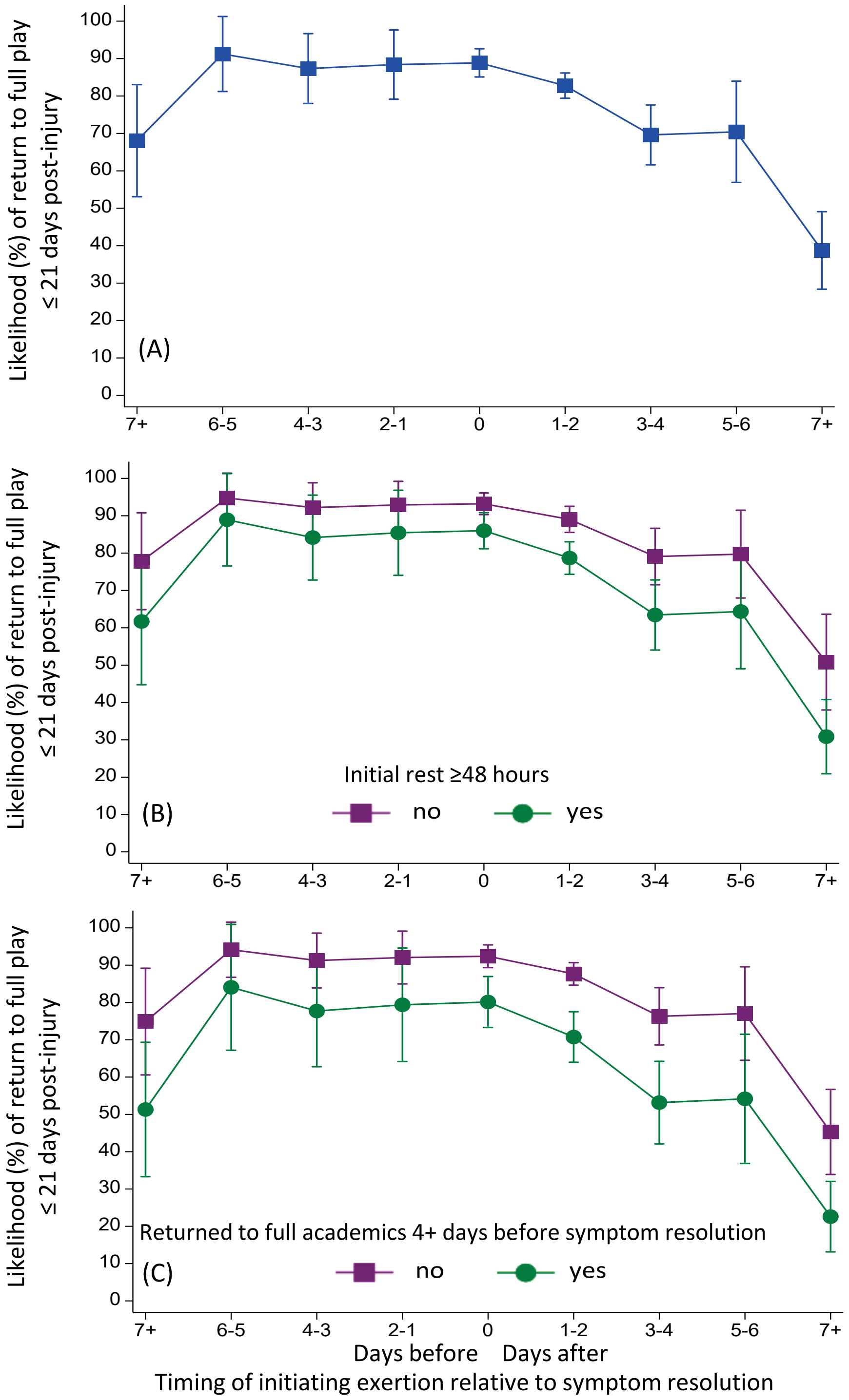

Estimated with logistic regression adjusting for athlete sex, number of symptoms, number of previous concussions, and returning to full academics before symptom resolution.
